# Supplementary figures and images for: Aggregated and Hyperstable Damage-Associated Molecular Patterns Are Released During ER Stress to Modulate Immune Function
Source: Front Cell Dev Biol. 2019 Sep 18;7:198. doi: 10.3389/fcell.2019.00198 (PMC6759876; doi:10.3389/fcell.2019.00198)

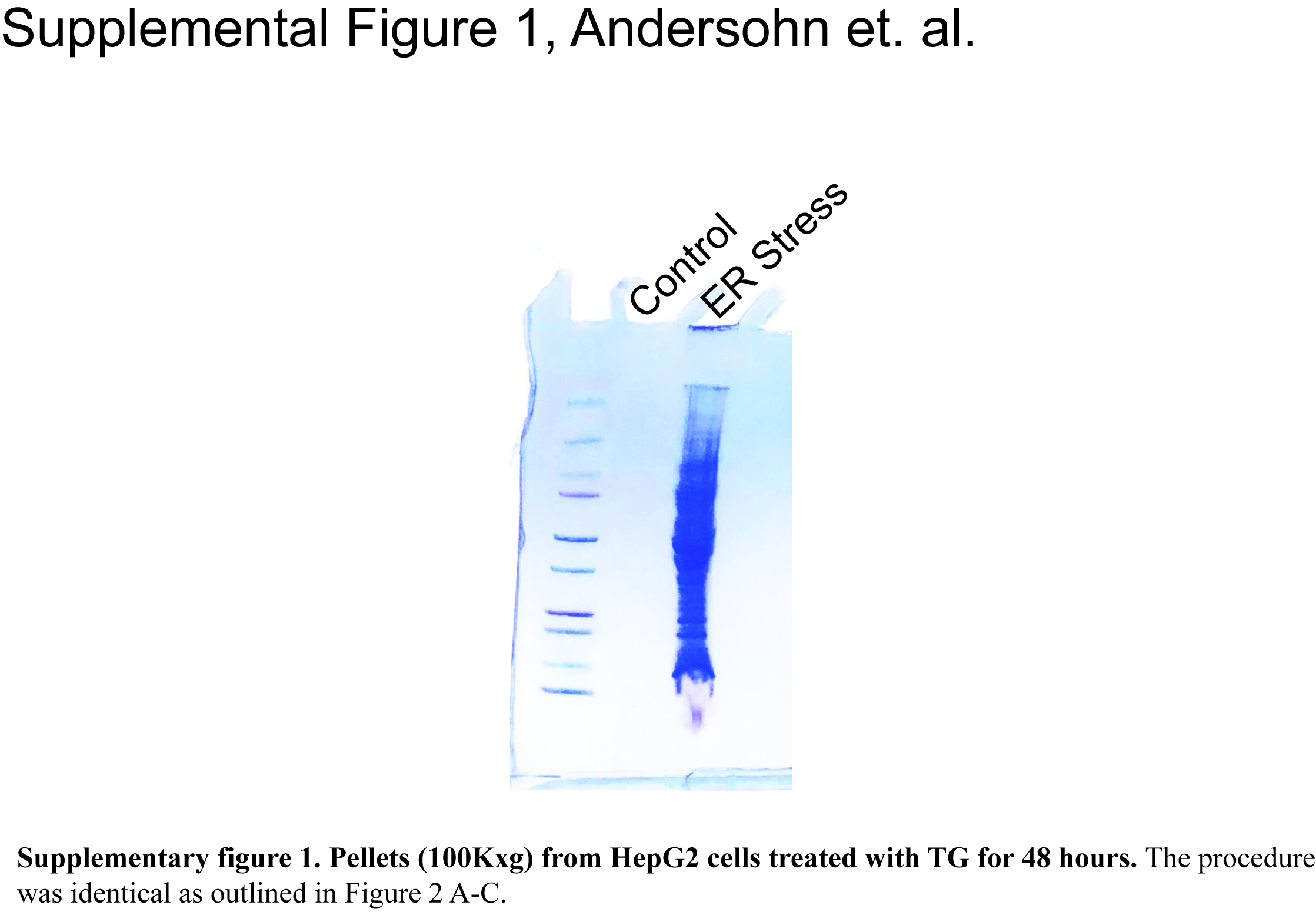

Supplement: Supplementary file 1 [file Image_1.TIF]

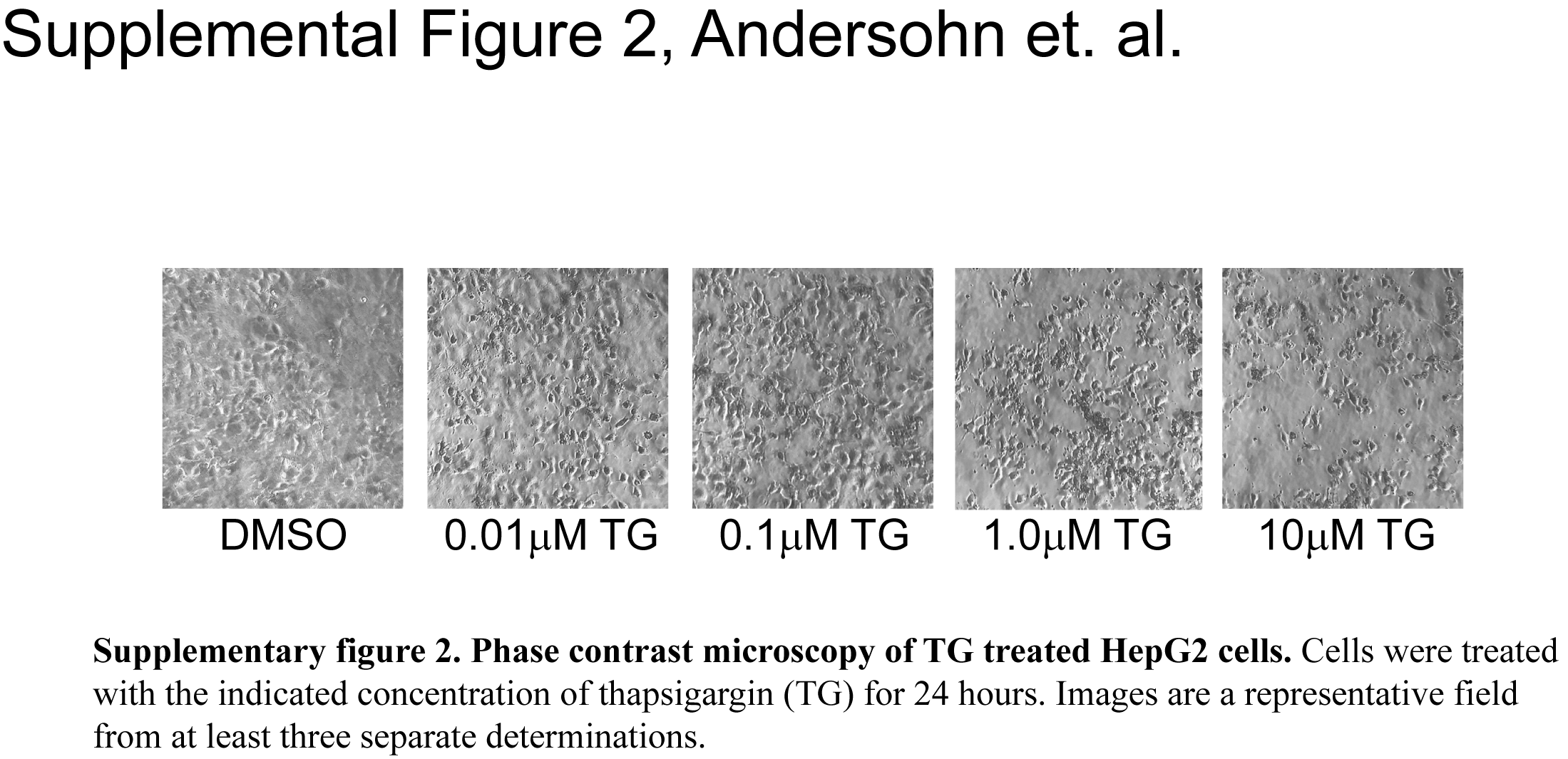

Supplement: Supplementary file 2 [file Image_2.TIF]

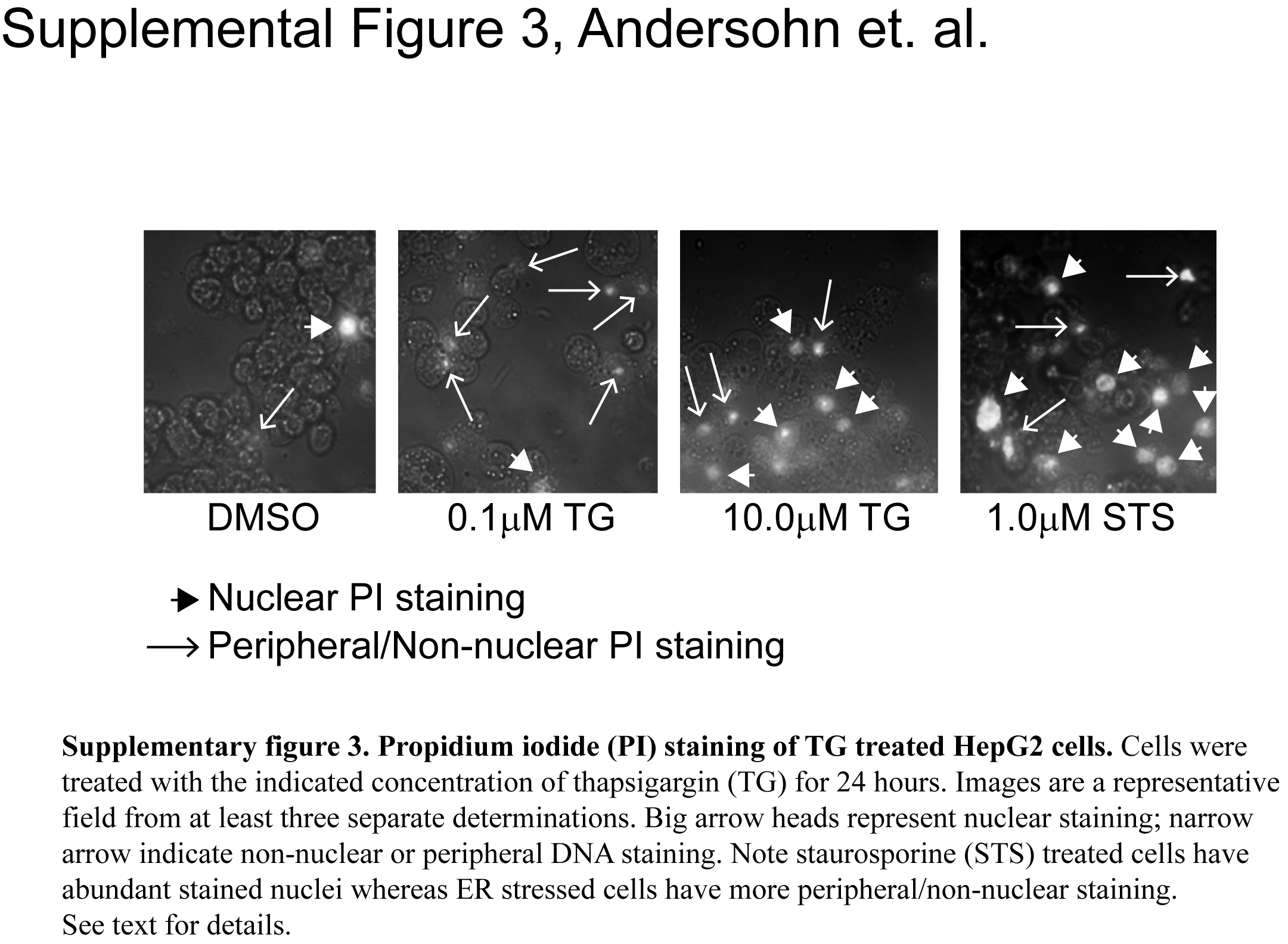

Supplement: Supplementary file 3 [file Image_3.TIF]

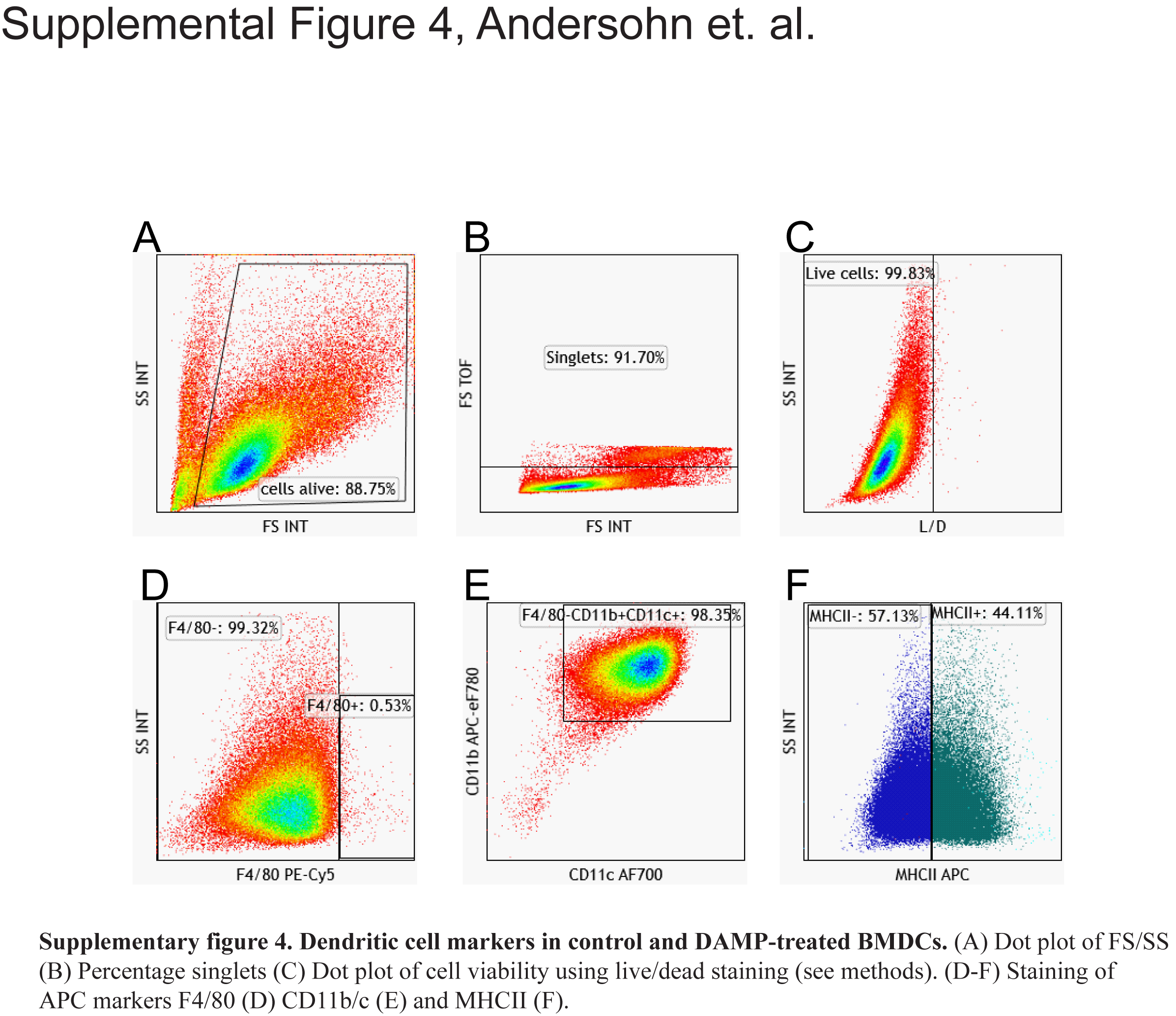

Supplement: Supplementary file 4 [file Image_4.TIF]

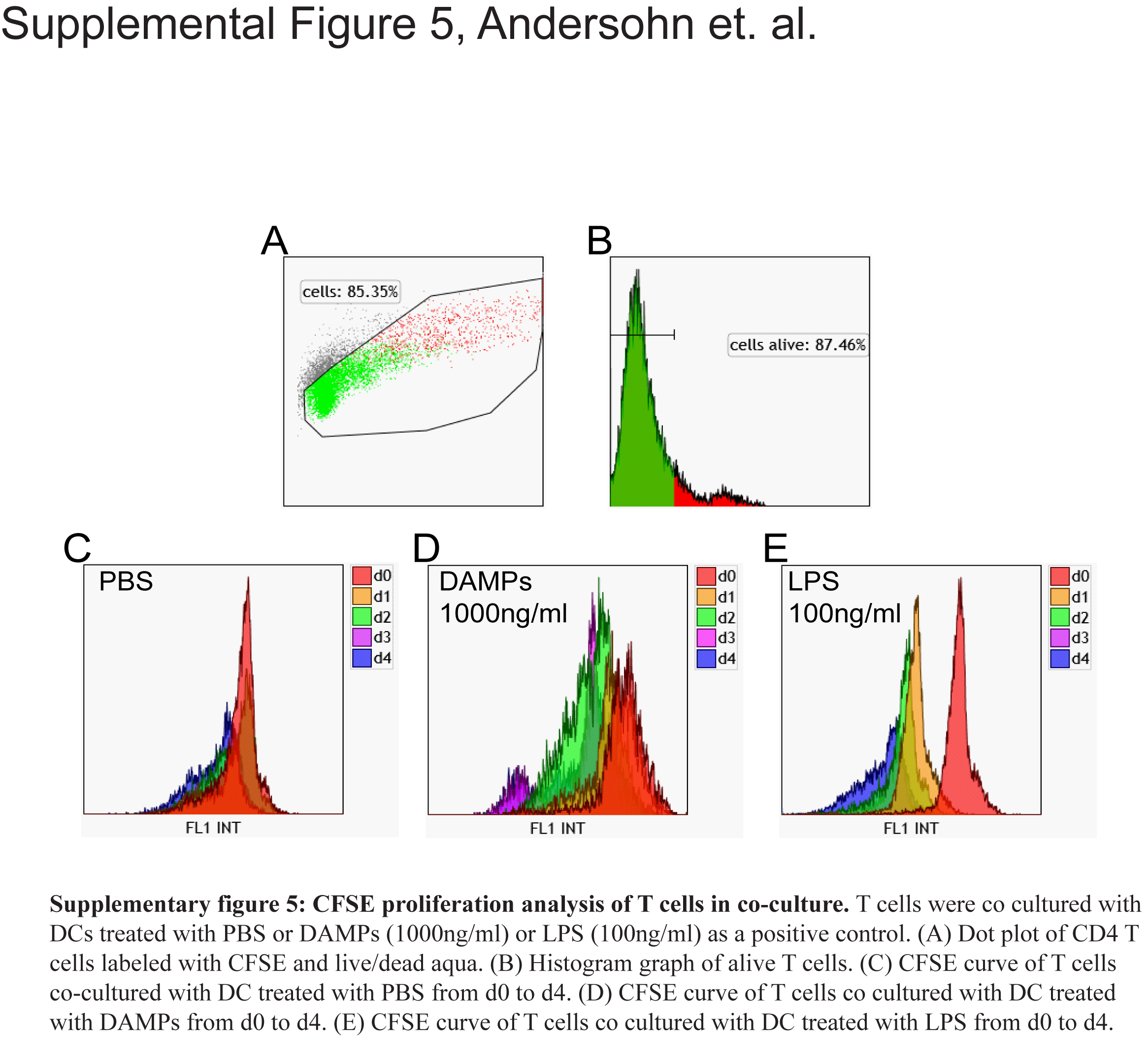

Supplement: Supplementary file 5 [file Image_5.TIF]

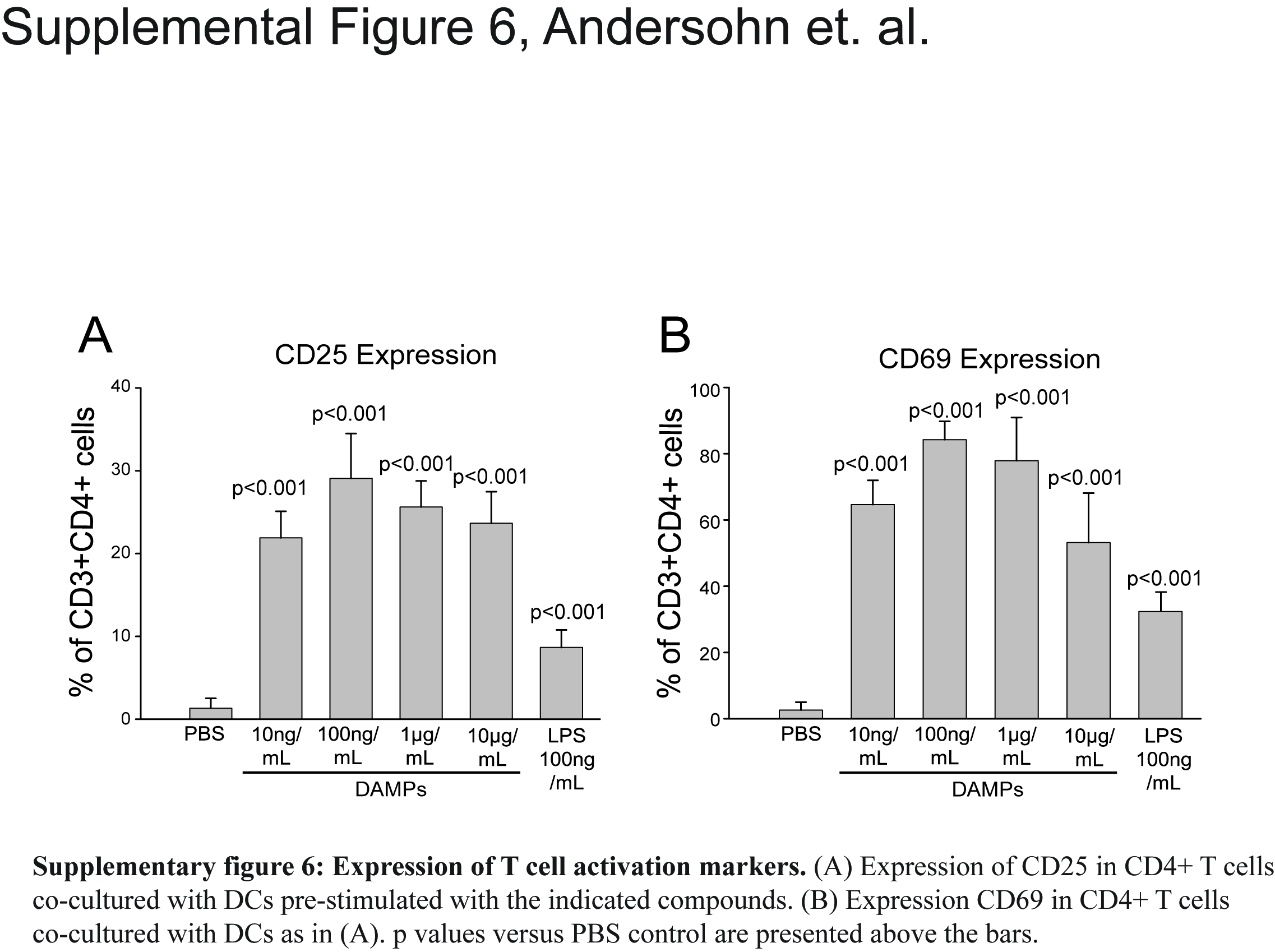

Supplement: Supplementary file 6 [file Image_6.TIF]

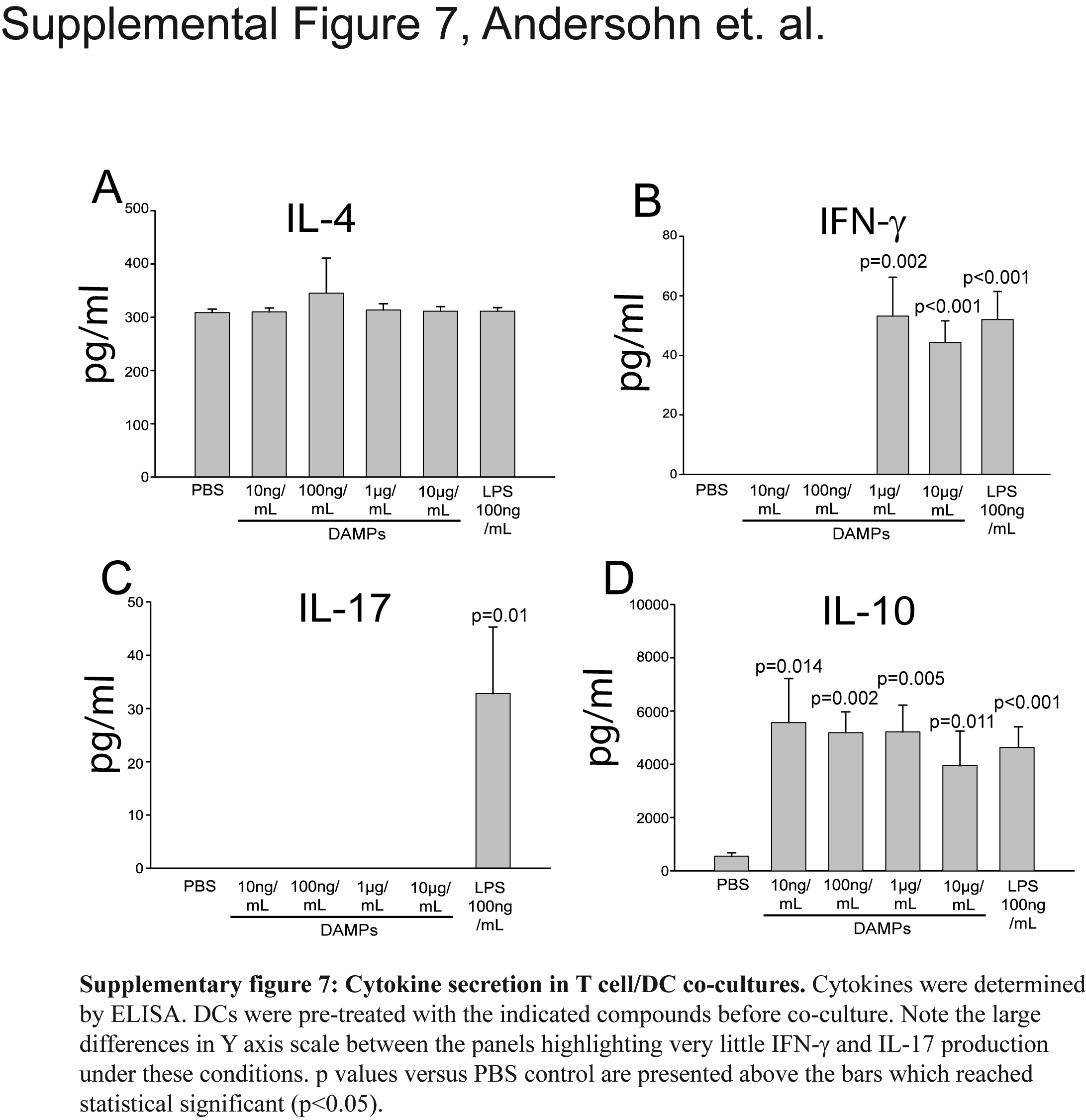

Supplement: Supplementary file 7 [file Image_7.TIF]
